# Discovering new peripheral plasma biomarkers to identify cognitive decline in type 2 diabetes

**DOI:** 10.3389/fcell.2022.818141

**Published:** 2022-11-24

**Authors:** Haitao Yu, Yang Gao, Ting He, Mengzhu Li, Yao Zhang, Jie Zheng, Bijun Jiang, Chongyang Chen, Dan Ke, Yanchao Liu, Jian-Zhi Wang

**Affiliations:** ^1^ Key Laboratory of Education Ministry of China/Hubei Province for Neurological Disorders, Department of Pathophysiology, School of Basic Medicine, Tongji Medical College, Huazhong University of Science and Technology, Wuhan, China; ^2^ Department of Basic Medicine, Wuxi School of Medicine, Jiangnan University, Wuxi, China; ^3^ Department of Neurosurgery, Wuhan Central Hospital Affiliated to Tongji Medical College, Huazhong University of Science and Technology, Wuhan, China; ^4^ Key Laboratory of Ministry of Education for Neurological Disorders, Li Yuan Hospital, Tongji Medical College, Huazhong University of Science and Technology, Wuhan, China; ^5^ Key Laboratory of Basic Pharmacology of Ministry of Education, Joint International Research Laboratory of Ethnomedicine of Ministry of Education, Key Laboratory of Basic Pharmacology of Guizhou Province, Department of Pharmacology, Zunyi Medical University, Zunyi, China; ^6^ Department of Physiology, School of Basic Medicine, Tongji Medical College, Huazhong University of Science and Technology, Wuhan, China; ^7^ Department of Neurosurgery, Tongji Hospital, Tongji Medical College, Huazhong University of Science and Technology, Wuhan, China; ^8^ Co-innovation Center of Neuroregeneration, Nantong University, Nantong, China

**Keywords:** type 2 diabetes, mild cognitive impairment, Alzheimer’s disease, diagnostic biomarkers, proteomics

## Abstract

Type 2 diabetes mellitus (T2DM) is an independent risk factor of Alzheimer’s disease (AD), and thus identifying who among the increasing T2DM populations may develop into AD is important for early intervention. By using TMT-labeling coupled high-throughput mass spectrometry, we conducted a comprehensive plasma proteomic analysis in none-T2DM people (Ctrl, *n* = 30), and the age-/sex-matched T2DM patients with mild cognitive impairment (T2DM-MCI, *n* = 30) or T2DM without MCI (T2DM-nMCI, *n* = 25). The candidate biomarkers identified by proteomics and bioinformatics analyses were verified by ELISA, and their diagnostic capabilities were evaluated with machine learning. A total of 53 differentially expressed proteins (DEPs) were identified in T2DM-MCI compared with T2DM-nMCI patients. These DEPs were significantly enriched in multiple biological processes, such as amyloid neuropathies, CNS disorders, and metabolic acidosis. Among the DEPs, alpha-1-antitrypsin (SERPINA1), major viral protein (PRNP), and valosin-containing protein (VCP) showed strong correlation with AD high-risk genes APP, MAPT, APOE, PSEN1, and PSEN2. Also, the levels of PP2A cancer inhibitor (CIP2A), PRNP, corticotropin-releasing factor-binding protein (CRHBP) were significantly increased, while the level of VCP was decreased in T2DM-MCI patients compared with that of the T2DM-nMCI, and these changes were correlated with the Mini-Mental State Examination (MMSE) score. Further machine learning data showed that increases in PRNP, CRHBP, VCP, and rGSK-3β(T/S9) (ratio of total to serine-9-phosphorylated glycogen synthase kinase-3β) had the greatest power to identify mild cognitive decline in T2DM patients.

## Introduction

Alzheimer’s disease (AD) is the most common form of senile dementia affecting an increasing population worldwide (Winblad et al., 2016; Hodson, 2018). Until most recently, there is still no effective cure for AD. One of the bottlenecks leading to the failed drug development is the lack of effective and non-brain invasive biomarkers for the early diagnosis of AD. The major dilemma is that it is often too late for an effective cure by the time the patients begin to feel AD symptoms, while the patients generally do not seek medical attention when they have no obvious AD symptoms. To deal with the dilemmas, one can start from AD high-risk factors to achieve its early diagnosis.

Type 2 diabetes mellitus (T2DM) is an independent risk factor of AD, and the latter is thus termed as type 3 diabetes (Diniz Pereira et al., 2021). Due to population aging, nutrition excess, lack of physical exercise, overweight, and other factors (Kahn et al., 2014), the number of T2DM patients is rapidly increasing and can greatly contribute to the increased prevalence of AD (Surguchov, 2020). Therefore, identifying biomarkers to predict who among the T2DM patients may develop into AD is important for the early diagnosis and eventually decreasing the prevalence of AD.

The mild cognitive impairment (MCI) is an intermediate state between normal aging and AD (Umegaki, 2014); therefore, it provides a window period for AD intervention and prevention (Petersen et al., 2001; Petersen et al., 2009). Aimed at seeking periphery biomarkers correlated to a very mild cognitive decline in T2DM patients, we used Mini-Mental State Examination (MMSE) to divide the T2DM patients into two cohorts: T2DM with MCI (T2DM-MCI) and T2DM without MCI (T2DM-nMCI). Using hypothesis-driven screening tests, we found that aging, upregulation of glycogen synthase kinase 3β (GSK-3β), a key kinase involved in Aβ production, tau hyperphosphorylation, and long-term synaptic inhibition seen in both AD and T2DM (Plattner et al., 2006; Yi et al., 2018), ApoE ε4 genetype, and olfactory dysfunction contributed to cognitive decline in T2DM patients (Xu et al., 2016). In addition, amyloidosis is a common pathological feature of AD and diabetes mellitus (de Matos et al., 2018), and the diagnostic accuracy of plasma Aβ1-42/1-40 combined with APP 669-711/Aβ1-42 was 90% for AD (Nakamura et al., 2018), but the potential association between plasma Aβ levels and cognitive functions has not been reported.

The memory impairment in AD and T2DM involves a complex network regulation of insulin resistance, oxidative stress, β-amyloid (Aβ) deposition, glucose and lipid metabolism, and vascular damage (Akter et al., 2011; de Matos et al., 2018). Therefore, non-hypothesis-driven approaches are needed for finding novel periphery biomarkers. In this sense, proteomic technology based on mass spectrometry has shown its strong role in the neurological field, such as overall analysis of protein expression levels, inter-molecular correlations, and biomarker screening (Bader et al., 2020; Wang et al., 2020). Peripheral blood is easy to obtain, and its dynamic changes can reflect the overall health situation of the individual (Anderson and Anderson, 2002), which makes it an ideal source of biomarkers to predict the changes occurred in the central nervous system. Compared with traditional 2D-DIGE, TMT/iTRAQ has the advantages of short cycle, small sample loss, simple operation, high sensitivity, and wide identification range. By using proteomic analysis, we have analyzed the platelet biomarkers correlated to cognitive decline in age- and sex-matched populations (Yu et al., 2021).

In the present study, we analyzed the plasma protein expression profile in T2DM-MCI and T2DM-nMCI patients by using high-throughput and highly sensitive TMT–LC–MS/MS proteomics. Further comprehensive bioinformatics, machine learning and biochemical analyses revealed that multiple proteins were involved in the cognitive changes in T2DM patients. Among them, the increases in PRNP, CRHBP, VCP, and rGSK-3β(T/S9) had the greatest power to identify cognitive decline in T2DM patients.

## Materials and methods

### Participant information

Type 2 diabetes diagnosis standard adopts the World Health Organization (WHO) (1999) criteria for diagnosis and classification of diabetes (Olokoba et al., 2012). All the type 2 diabetes mellitus (T2DM) patients from the Central Hospital of Wuhan were divided into two groups: the T2DM without mild cognitive impairment (T2DM-nMCI) group and the T2DM with mild cognitive impairment (T2DM-MCI) group, which met the National Institute on Aging and the Alzheimer’s Association Guidelines (Albert et al., 2011), and received Mini-Mental State Examination (MMSE) test scores (Folstein et al., 1975) (Table 1). Patients with traumatic brain injury, brain tumors, drug abuse, alcohol addiction, and psychiatric disorders were excluded from the study. Diabetes duration, hypertension, hyperlipidemia and coronary heart disease (CHD), olfactory score, and Apo lipoprotein E (APOE) were considered systematically (Table 1).

**TABLE 1:** Information about the T2DM-MCI patients and age-/sex-matched T2DM-nMCI patients.

Characteristic	Proteomics-Discover set			ELISA-Validation set		
	T2DM-nMCI (*n* = 25)	T2DM-MCI (*n* = 30)	*p*-value	T2DM-nMCI (*n* = 30)	T2DM-MCI (*n* = 25)	*p*-value
Age, mean (SD), year	70.68 (5.31)	76.27 (5.31)	<0.001	72.27 (2.52)	73.33 (4.20)	0.237
Sex (male, female)	15M, 10F	8M, 22F	0.016	14M, 16F	14M, 16F	>0.999
Olfactory score	7.78 (1.49)	8.941.49	0.026	7.43 (1.57)	7.87 (1.48)	0.276
Diabeted = s duration, year	11.65 (7.18)	7.87 (6.88)	0.086	11.87 (8.45)	11.83 (9.17)	0.988
Hypertension, n (%)	15 (75.00%)	19 (82.61%)	0.711	23 (76.67%)	26 (86.67%)	0.317
Hyperlipidemia, n (%)	2 (10.00%)	8 (34.78%)	0.076	3 (10.00%)	13 (44.33%)	0.004
CHD, n (%)	4 (20.00%)	2 (8.70%)	0.393	7 (23.33%)	7 (23.33%)	>0.999
APOE ε 4 (+), n (%)	4 (20.00%)	4 (17.39%)	>0.999	4 (13.33%)	4 (13.33%)	>0.999
GSK-3 β (S9)	1.77 (0.60–3.57)	1.28 (0.11–3.17)	0.062	1.92 (0.53–14.60)	1.55 (0.48–5.59)	0.484
GSK-3 β (total)	1.03 (0.13–2.38)	1.42 (0.11–3.17)	0.058	1.22 (0.13–7.16)	2.22 (0.63–10.64)	0.03
RGSK- 3 β (total/S9)	0.63 (0.18–1.66)	1.13 (0.24–2.48)	<0.01	0.67 (0.20–1.55)	2.31 (0.59–17.14)	0.011
Aβ 1–40	282.4 (64.10–590.50)	211.80 (22.89–430.70)	0.110	239.78 (14.81–520.01)	189.47 (15.07–559.41)	0.174
Aβ 1–42	66.07 (31.99–153.60)	78.68 (37.58–162.90)	0.170	54.13 (20.22–100.82)	78.89 (32.47–172.87)	0.002
Aβ 1–42/1–40	0.30 (0.07–063)	0.70 (0.15–3.68)	<0.05	0.41 (0.07–4.37)	1.23 (0.08–7.60)	0.018
MMSE	30 (0)	18.63	<0.001	28.93	21.67 (1.95)	<0.001

The study was approved by the Tongji Medical School Ethics Committee, complies with the Helsinki Declaration II, and includes written informed consent from all participants. The project “Early Detection of Cognitive Dysfunction in Diabetes” was registered in the Chinese Clinical Trial Registry (https://clinicaltrials.gov; NCT01830998).

Fresh blood was stored in an anticoagulant tube, and the blood sample is separated at 1,000 g, 4°C for 10 min within 2 h to separate plasma and stored at −80°C. According to Agilent Technologies operating instructions (Yadav et al., 2011), we used Agilent Human 14/Mouse 3 Multiple Affinity Removal System Column to remove high-abundance proteins in plasma. Then, for desalination and concentration of low-abundance components, we used a 10-kDa ultrafiltration tube (Sartorius) and added SDT buffer (4% SDS, 100 mM DTT, and 150 mM Tris–HCl pH 8.0) to the sample, boiled for 15 min, and centrifuged at 14,000 g for 20 min. The BCA method was used for protein quantification.

### Filter-aided sample preparation

The FASP digestion was carried out by following a previous method (Wisniewski et al., 2009). Specifically, 200 μg of protein solution for each sample was added to dithiothreitol (DTT, Sigma-Aldrich) to obtain a final concentration of 100 mM and boiled in a water bath for 5 min. Then, 200 μl UA buffer (8 M Urea and 150 mM Tris–HCl, pH 8.0) was added to the mixture, centrifuged at 14000 *g* for 15 min in a 10-kD ultrafiltration centrifuge tube, and incubated with 100 mM iodoacetamide (IAA, Sigma-Aldrich) at room temperature for 30 min in darkness. Then, the aforementioned samples were washed twice with 100 ul UA buffer and 100 ul 100 mM TEAB buffer. Finally, digestion was carried out with 4 μg trypsin (Promega) in 40 μl TEAB buffer for 12 h at 37°C, and the peptide concentration was determined using a NanoDrop 2,000c spectrophotometer (Thermo Fisher Scientific, Waltham, MA, United States).

### Tandem mass tag labeling and separation

The peptides (100 μg) were labeled with TMT tags according to the manufacturer’s instructions (Thermo Fisher Scientific). To fractionate TMT-labeled peptides, high-pH reversed-phase chromatography separation was used. According to the manufacturer’s instructions (Thermo Fisher Scientific), TMT-labeled peptides were divided into 15 components under gradient elution. After lyophilization, the sample was reconstituted with 12 μl of 0.1% FA, and the peptide concentration was determined using a NanoDrop 2,000c spectrophotometer.

### Mass spectrometry data collection

The peptide mixture was processed on-machine for nanoLC-MS/MS analysis. Buffer A is 0.1% formic acid aqueous solution, and buffer B is 0.1% formic acid acetonitrile aqueous solution (84% acetonitrile). The chromatographic column was equilibrated with 95% buffer A (0.1% formic acid), and the sample was loaded from the autosampler to the upper column (Thermo Scientific Acclaim PepMap100, 100 μm* 2cm, nanoViper C18) after separation by the analytical column (Thermo Scientific Easy Column, 10 cm long, 75 μm inner diameter, 3 μm resin) with buffer B (84% acetonitrile and 0.1% formic acid) at a flow rate of 300 nl/min.

To acquire MS data, the criteria used were as follows: running in the positive ion mode, the scanning range of parent ion was 300–1800 m/z, the resolution of primary mass spectrometry was 70,000 at 200 m/z, and isolation width was 2 m/z with 30 ev standardized collision energy. MS/MS spectra were searched using MASCOT engine (Matrix Science, London, United Kingdom, version 2.2) with Proteome Discoverer 1.4. The value of the protein ratio ≥ 1.2 with the *p*-value < 0.05 was defined as the differential protein.

### Bioinformatics analysis

The heatmap.2 function in the R statistical analysis software package (version 3.4.0) was used to perform hierarchical clustering analysis for differential proteins. DAVID Bioinformatics Resources 6.7 (https://david-d.ncifcrf.gov/) was used for biological process analysis. Metascape (http://metascape.org/gp/) was used for protein–protein interaction (PPI) and gene–disease network (DisGeNET) analysis.

### Enzyme-linked immunosorbent assay

The plasma levels of Aβ42/40, CIP2A, PRNP, CRHBP, VCP, IGFBP3, and PEPD in T2DM-nMCI and T2DM-MCI were verified by ELISA following the manufacturer’s instructions (Table 2).

**TABLE 2:** List of ELISA kit used in the study.

Specificity	Source	CAT. No.
Insulin-like growth factor-binding protein 3(IGFBP3)	Cloud-Clone	SEA054Hu
Corticotropin-releasing hormone-binding protein (CRHBP)	Cloud-Clone	SEC401Hu
Prion protein (PRNP)	Cloud-Clone	SEB680Hu
Cancerous inhibitor of PP2A (CIP2A)	Cloud-Clone	SER982Hu
Valosin-containing protein (VCP)	Cloud-Clone	SEC601Hu
Peptidase d (PEPD)	Cloud-Clone	E-EL-H0542c
Amyloid beta 1–40 (A β .1.40 )	Elabscience	E-EL-H0543c
Amyloid beta 1–42 (A β .1.42 )	Elabscience	

### Machine learning

The leave-one-out (LOO) cross-validation with the scikit-learn Python package was performed on the validation set, and AUC and accuracy values were calculated. As a typical machine learning method, the LOO cross-validation is widely used in life sciences for model training and parameter optimization (Bader et al., 2020; Shu et al., 2020). More specifically, 59 samples were randomly selected from 60 samples each time for modeling, and the remaining one was used for validation. Thus, 60 cycles were carried out to achieve the purpose of full data demonstration and cross-validation.

### Statistical analyses

The data were expressed as mean ± SEM using SPSS 24.0 software (Statistical Program for Social Sciences Inc., Chicago, IL, United States). Student’s t-test was used to evaluate the level of significance between the two groups, and *p-values* < 0.05 was considered to be significant.

## Results

### Participant information and deep plasma proteome profiling in different populations

In the discovery cohort, the age- and sex-matched none-T2DM controls (Ctrl, *n* = 30), T2DM-nMCI (*n* = 25), and T2DM-MCI (*n* = 30) were analyzed. The candidate biomarkers were further verified in the validation cohorts of T2DM-nMCI (*n* = 30) and T2DM-MCI (*n* = 30) (Table 1).

**FIGURE 1:**
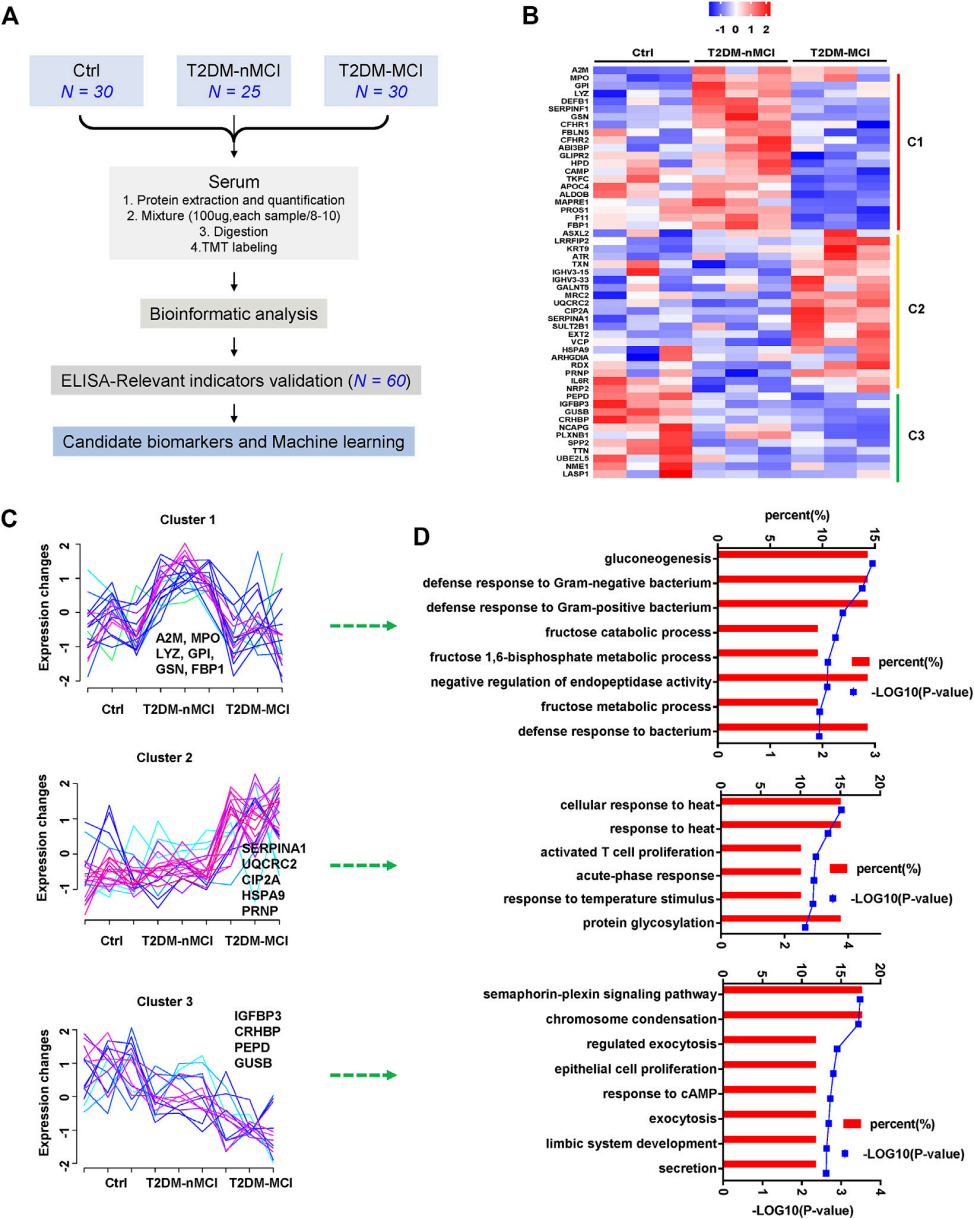
Disease stage-dependent proteins in the plasma of T2DM patients identified by proteomics. **(A)** Strategy for profiling of the serum proteome and subsequent verification of candidate biomarkers. **(B)** Heatmap of the proteomics dataset based on the changed proteins. The TMT intensities (Z-score transformed) of each protein (rows) across three disease groups (columns) are indicated in a colored scale. **(C)** Clustering analysis of changed proteins. Each line represents the change trend of one protein. The intensity of each protein is Z-score-transformed. **(D)** Enrichment analysis of biological processes for each cluster protein.

Plasma proteomics and the ELISA verification were combined to find candidate biomarkers for cognitive decline in T2DM patients (Figure 1A). A total of 1,320 proteins were identified in plasma proteomics, of which 752 proteins were captured in each sample. The differentially expressed proteins (DEPs) were defined as the fold change of protein abundance at 1.2 or above with the *p-value* < 0.05. In this study, from Ctrl to T2DM-nMCI and T2DM-MCI groups, a total of 53 DEPs were identified, including 22 DEPs in T2DM vs. Ctrl and 36 DEPs in T2DM-MCI vs. T2DM-nMCI (Figure 1B). After Z-score transformation, the abundance of DEPs was displayed in the form of a heatmap, and the DEPs were divided into three clusters (C1, C2 and C3; Figures 1B,C).

Cluster 1 displays a significant increase in T2DM vs. Ctrl but a decrease in T2DM-MCI vs. T2DM-nMCI (Figure 1C), including immune response-related proteins alpha-2-macroglobulin (A2M), myeloperoxidase (MPO), gelsolin (GSN), gluconeogenesis/glycolysis-related proteins glucose-6-phosphate isomerase (GPI), and fructose-1,6-bisphosphatase 1 (FBP1) (Figure 1D). The proteins displayed by cluster 2 were stable in T2DM vs. Ctrl but were significantly up-regulated in T2DM-MCI vs. T2DM-nMCI (Figure 1C), including alpha-1-antitrypsin (SERPINA1), PP2A cancer inhibitor (CIP2A), major prion protein (PRNP), mitochondria-related proteins cytochrome b-c1 complex subunit 2 (UQCRC2), valosin-containing protein (VCP), and stress-70 protein (HSPA9), involving cellular response to the heat stress biological process (Figure 1D). The proteins displayed by cluster 3 were significantly down-regulated from Ctrl to T2DM-nMCI and T2DM-MCI (Figure 1C), including learning- or memory-related proteins insulin-like growth factor-binding protein 3 (IGFBP3), beta-glucuronidase (GUSB), and corticotropin-releasing factor-binding protein (CRHBP), involving the secretion process (Figure 1D).

These data together indicate that multiple pathways and proteins are involved in cognitive decline in T2DM patients compared with the non-T2DM control group.

### Integrating analyses to identify human disease-associated differentially expressed proteins

**FIGURE 2:**
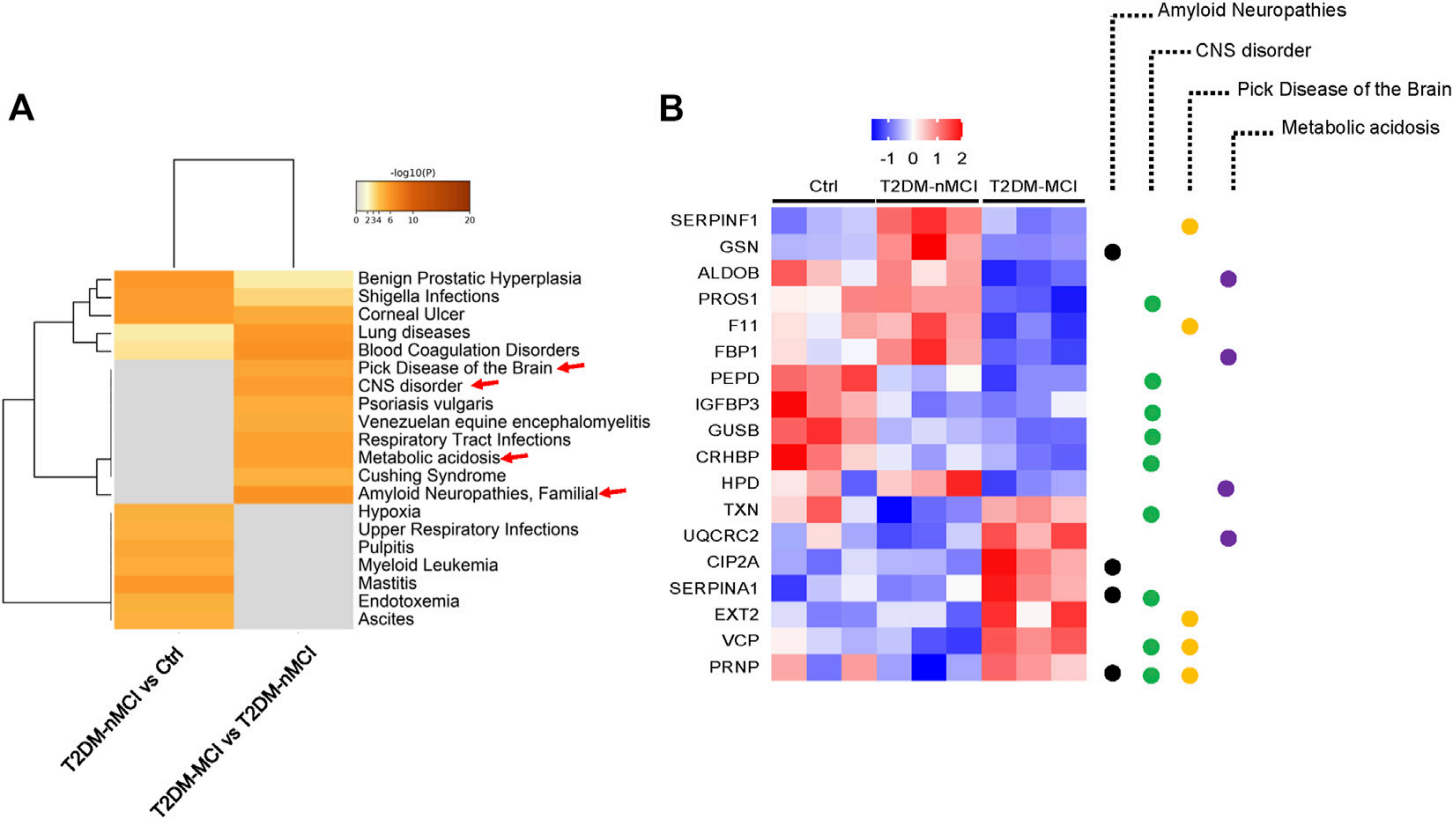
Integrating information on human disease-associated DEPs analyzed using Metascape. **(A)** Top GO terms of the gene–disease network (DisGeNET) with -log10 (*p-value* < 0.01). **(B)** Relative expression abundance of amyloid neuropathies, CNS disorder, Pick’s disease, and metabolic acidosis (*p* < 0.05, increased proteins: red; decreased proteins: blue).

To explore the relationship between DEPs and human diseases, we performed a gene–disease network (DisGeNET) analysis using Metascape online analysis software. Interestingly, the central system-related GO terms including “amyloid neuropathies,” “CNS disorder,” and “pick disease of the brain” were strongly enriched in T2DM-MCI vs T2DM-nMCI (*p-value* < 0.01) (Figure 2A). In addition, metabolic acidosis was also found in T2DM-MCI subjects compared with T2DM-nMCI (Figure 2A). Specifically, the proteins associated with the central system, including the well-known PRNP, CIP2A, fructose-bisphosphate aldolase B (ADLOB), and fructose-1,6-bisphosphatase 1 (FBP1) and the less-known SERPINA1 and VCP, were increased in T2DM-MCI compared with T2DM-nMCI, in which ADLOB and FBP1 were involved in metabolic acidosis (Figure 2B).

### The candidate proteins correlated with cognitive decline in T2DM patients

**FIGURE 3:**
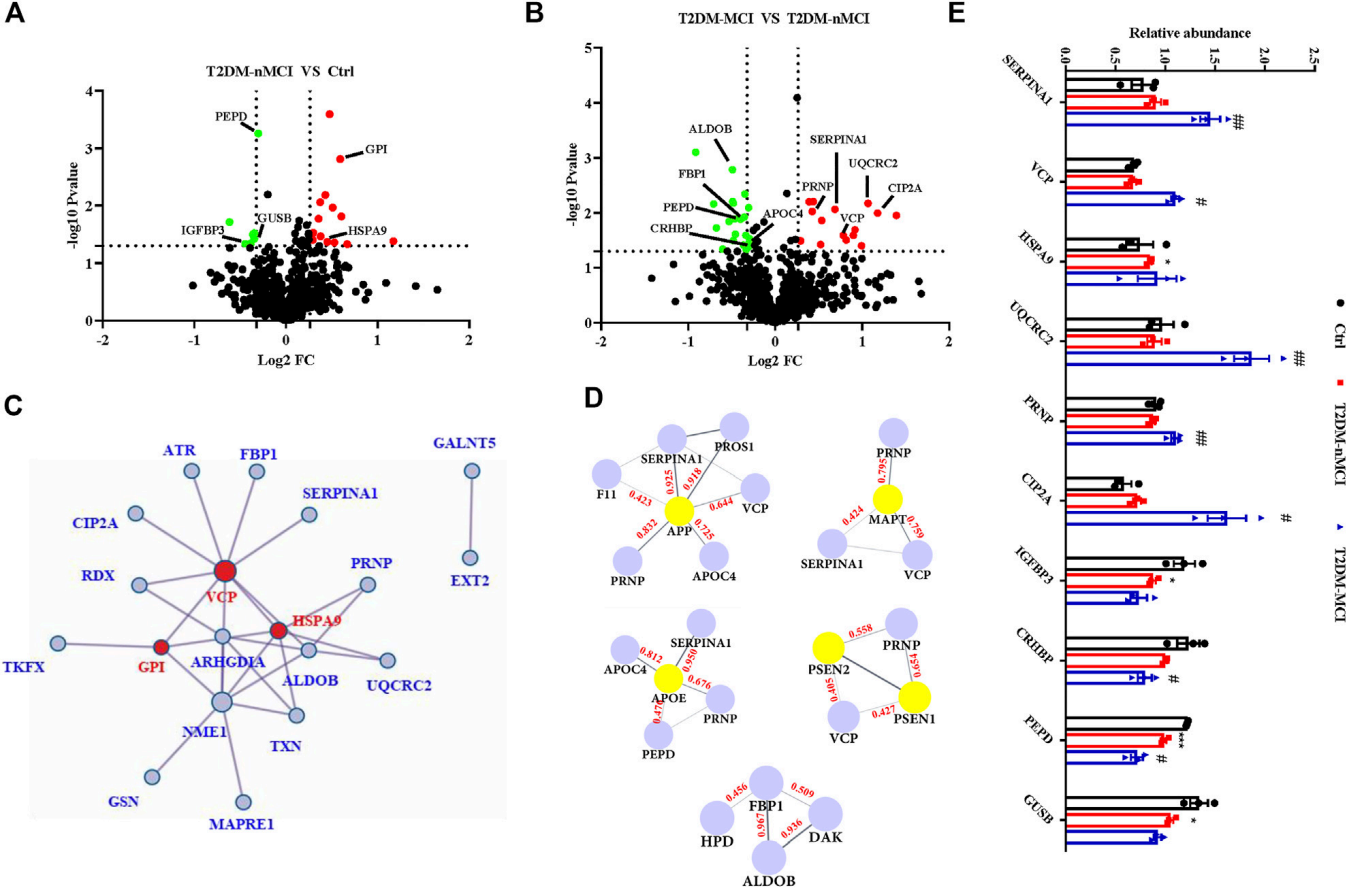
Protein–protein interaction network of DEPs in T2DM-MCI patients analyzed by a comprehensive analysis. **(A,B)** Volcano plot for the identified plasma proteins in T2DM-nMCI vs. Ctrl and T2DM-MCI vs. T2DM patients. Red and green dots indicate significantly up-regulated and down-regulated proteins, respectively. **(C)** All the differentially expressed proteins (DEPs) were visualized and mapped using Cytoscape 3.7.1. STRING analyses of hippocampal DEPs. Red dots indicate key regulatory nodes. **(D)** PPI modules associated with AD high-risk genes. Based on existing database experiments and data, STRING online analysis was used to obtain APP, MAPT, APOE, PSEN1/2, and DE protein–protein interaction modules (interaction score >0.4). **(E)** Relative expression levels of key proteins in T2DM-nMCI and T2DM-MCI. Data are presented as mean ± SEM. *p < 0.05, **p < 0.01, and ***p < 0.001 vs. Ctrl group; ^#^ *p* <0.05 and ^##^ *p* <0.01 vs. T2DM-nMCI group.

Compared with the non-T2DM control group, 22 significant dysregulated proteins were identified in T2DM-nMCI patients, including 14 up-regulated proteins (red) and eight down-regulated proteins (green) (Figure 3A, (Supplementary Table S1). When compared with T2DM-nMCI patients, 36 significant dysregulated proteins were identified in T2DM-MCI patients, including 15 up-regulated proteins (red) and 21 down-regulated proteins (green) (Figure 3B, Supplementary Table S1). The combination of Cytoscape (3.7.0) and Metascape was used to analyze the protein–protein interaction (PPI) network. As shown in Figure 3C, VCP, stress-70 protein (HSPA9), and GPI were at the core of the entire network. Importantly, PPI interaction analysis showed that dysregulated proteins SERPINA1, VCP, APOC4, and PRNP had strong interactions with AD high-risk genes APP, MAPT, APOE, PSEN1/2 (Figure 3D).

The bioinformatics data together indicate that upregulation of SERPINA1, VCP, PRNP, CIP2A, HSPA9, and UQCRC2 and downregulation of IGFBP3, CRHBP, PEPD, and GUSB were correlated to cognitive decline in T2DM patients (Figure 3E). The increased candidate proteins also include: 1) alpha-1-antitrypsin (SERPINA1), which is responsible for serine-type endopeptidase inhibitor activity; 2) transitional endoplasmic reticulum ATPase (VCP), a mitophagy-related protein that is mainly involved in frontotemporal dementia, amyotrophic lateral sclerosis, and muscle and bone degeneration (Kakizuka, 2008); 3) stress-70 protein (HSPA9), a mitochondria-related protein, as a risk factor for Parkinson’s disease (PD) and Alzheimer’s disease (AD) (Chung et al., 2017); 4) cytochrome b-c1 complex subunit 2 (UQCRC2), whose binding to Aβ leads to mitochondrial dysfunction (Nakamura et al., 2009); 5) major prion protein (PRNP) that participates in the neurotoxicity caused by Aβ; when it is knocked out, the synaptic defects of AD mice are rescued (Gimbel et al., 2010; Salazar et al., 2017); and 6) cancerous inhibitor of PP2A (CIP2A) that promotes AD pathology by inhibiting PP2A activity (Shentu et al., 2018). The decreased candidate proteins also include: 1) insulin-like growth factor-binding protein 3 (IGFBP3), which affects the course of AD by regulating the level of free IGF in the brain (Jogie-Brahim et al., 2009); 2) corticotropin-releasing factor-binding protein (CRHBP), which is involved in CRF-mediated hypothalamic–pituitary–adrenal (HPA) axis imbalance associated with AD under chronic stress (Vandael and Gounko, 2019); 3) xaa-Pro dipeptidase (PEPD), whose deficiency is associated with immune malfunction and intellectual disability (Kitchener and Grunden, 2012); and 4) beta-glucuronidase (GUSB), a biomarker related to memory and AD (Niculescu et al., 2019).

Collectively, these candidate proteins may have great potential for distinguishing T2DM-nMCI and T2DM-MCI.

### Validation of the candidate proteins by ELISA

**FIGURE 4:**
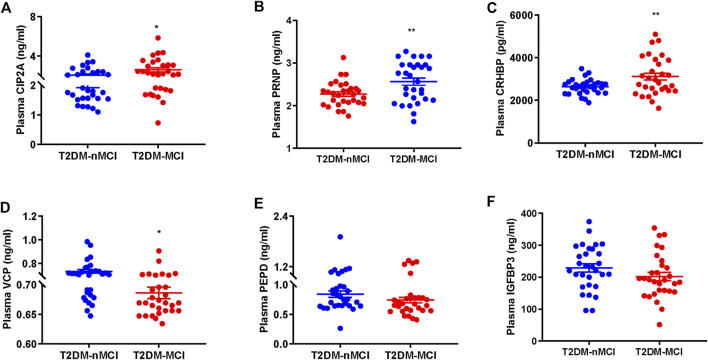
Validation of proteomics-identified plasma candidate biomarkers by ELISA. **(A–F)** Levels of plasma-derived CIP2A **(A)**, PRNP **(B)**, CRHBP **(C)**, VCP **(D)**, PEPD **(E)**, and IGFBP3 **(F)** in T2DM-nMCI and T2DM-MCI. *p < 0.05 and **p < 0.01 *vs*. T2DM-nMCI group.

**FIGURE 5:**
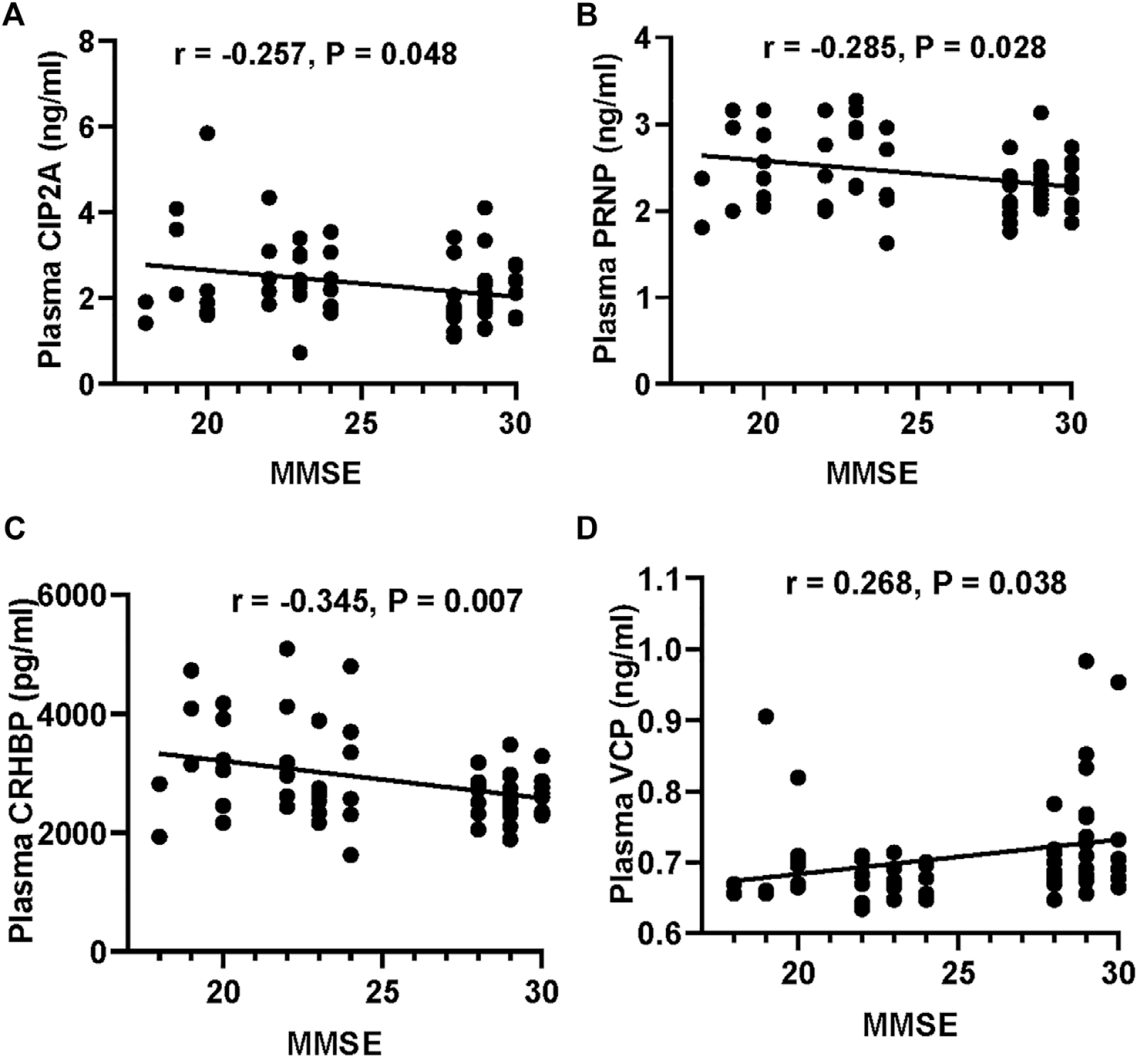
Correlation of the potential marker protein levels with MMSE. **(A–D)** Correlations between CIP2A or PRNP or CRHBP or VCP levels and MMSE scores.

We first validated the candidate proteins CIP2A, PRNP, VCP, CRHBP, IGFBP3, and PEPD in T2DM-nMCI (*n* = 30) and T2DM-MCI (*n* = 30) patients. Compared with T2DM-nMCI, the protein levels of CIP2A, PRNP, and CRHBP were significantly increased in T2DM-MCI, while the level of VCP was decreased (Figures 4A–D). Consistent with the proteomics results, PEPD and IGFBP3 showed a decreasing trend in a limited number of samples (Figures 4E,F). Using Pearson’s analysis, we found that changes of CIP2A (*r* = −0.257, *p* = 0.048, Figure 5A), PRNP (*r* = −0.285, *p* = 0.028, Figure 5B), CRHBP (*r* = −0.345, *p* = 0.007, Figure 5C), and VCP (*r* = 0.268, *p* = 0.038, Figure 5D) were correlated with the MMSE score.

**FIGURE 6:**
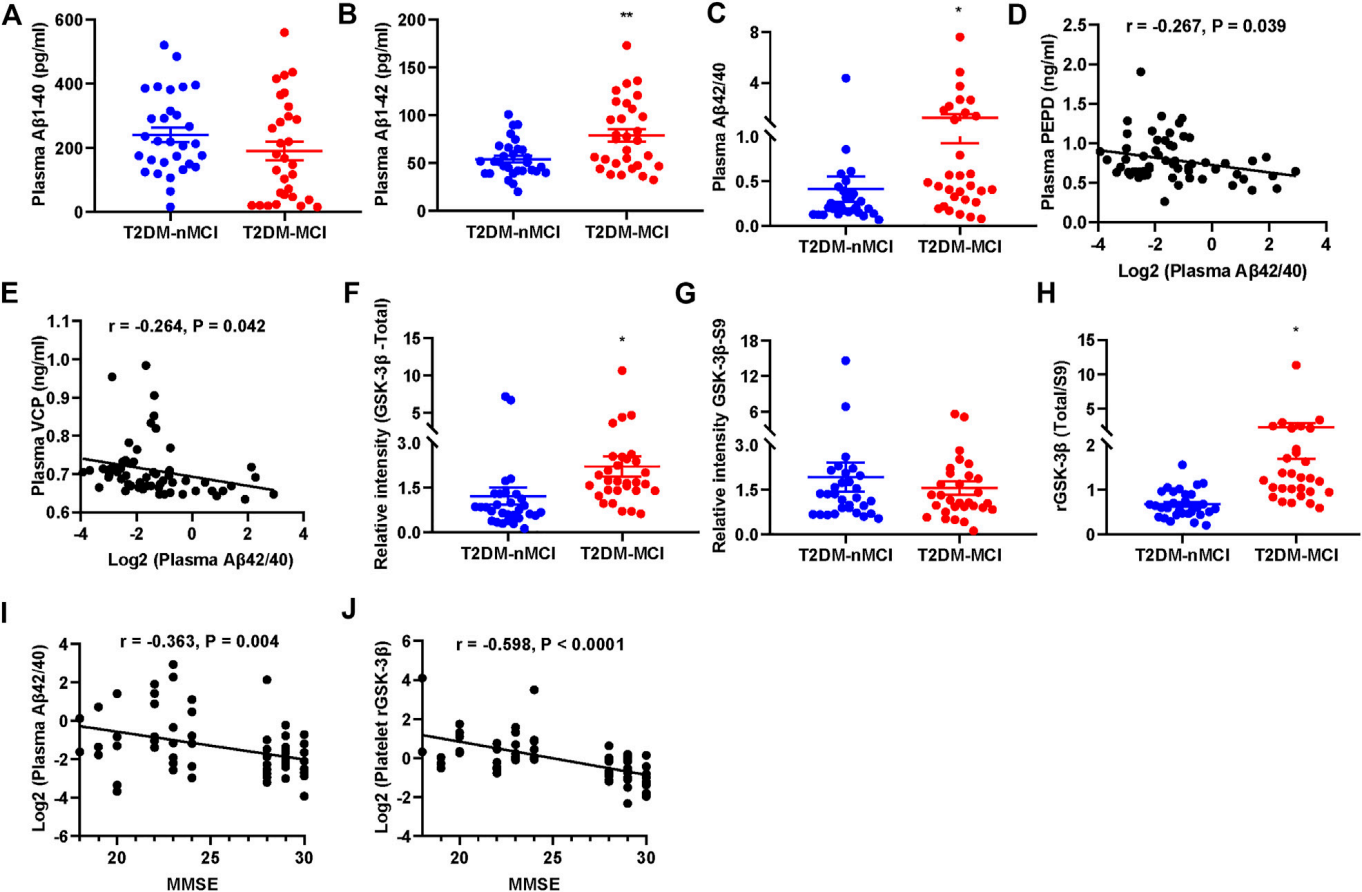
Elevated plasma Aβ42/40 and platelet rGSK-3β and their correlations with MMSE in T2DM-MCI patients. **(A–C)** Comparison of plasma Aβ1-40, Aβ1-42, and Aβ42/40 in T2DM-MCI *vs.* T2DM-nMCI. **(D,E)** Correlation of the PEPD or VCP level with the Aβ42/40 ratio. **(F–H)** Comparison of platelet GSK-3β-total, GSK-3β-S9, and rGSK-3β (total/S9) in T2DM-MCI *vs*. T2DM-nMCI. *p < 0.05 and **p < 0.01 *vs*. T2DM-nMCI subjects. **(I,J)** Correlation of Aβ42/40 or rGSK-3β with MMSE scores (Aβ42/40 and MMSE: *r* = −0.363, *p* = 0.004; rGSK-3β and MMSE: *r* = −0.598, *p* < 0.0001).

A previous report showed that the ratio of plasma Aβ42/40 was decreased in AD patients (Nakamura et al., 2018). We also detected the plasma Aβ levels in T2DM patients with or without MCI. We observed that the plasma level of Aβ42 and Aβ42/40 were significantly increased in T2DM-MCI compared with that of T2DM-nMCI patients, while no difference in Aβ40 was shown (*p* = 0.174, Figures 6A–C). Additionally, the changes in PEPD and VCP were negatively correlated with Aβ42/40, though the correlation coefficient was relatively low (PEPD: *r* = -0.267, *p* = 0.039; VCP: *r* =−0.264, *p* = 0.042, Figures 6D,E). An elevated platelet GSK-3β activity could discriminate T2DM-MCI from T2DM-nMCI (Xu et al., 2016). Here, we also observed that platelet GSK-3β-total and the ratio of GSK-3β-total to GSK-3β-S9 (rGSK-3β, activity) were significantly increased in T2DM-MCI compared with those of T2DM-nMCI patients, while no difference was observed in GSK-3β-S9 (inactive form, *p* = 0.484, Figures 6F–H). Both Aβ42/40 and rGSK-3β were moderately correlated with the MMSE score (Aβ42/40: *r* = −0.363, *p* = 0.004; rGSK-3β: *r* = −0.598, *p* < 0.001, Figures 6I,J), suggesting their association with cognitive decline.

These data together indicate that periphery CIP2A, PRNP, VCP, CRHBP, Aβ42/40, and rGSK-3β could be biomarkers for cognitive decline in T2DM patients.

### Machine learning shows diagnostic efficacy of the potential biomarkers

**FIGURE 7:**
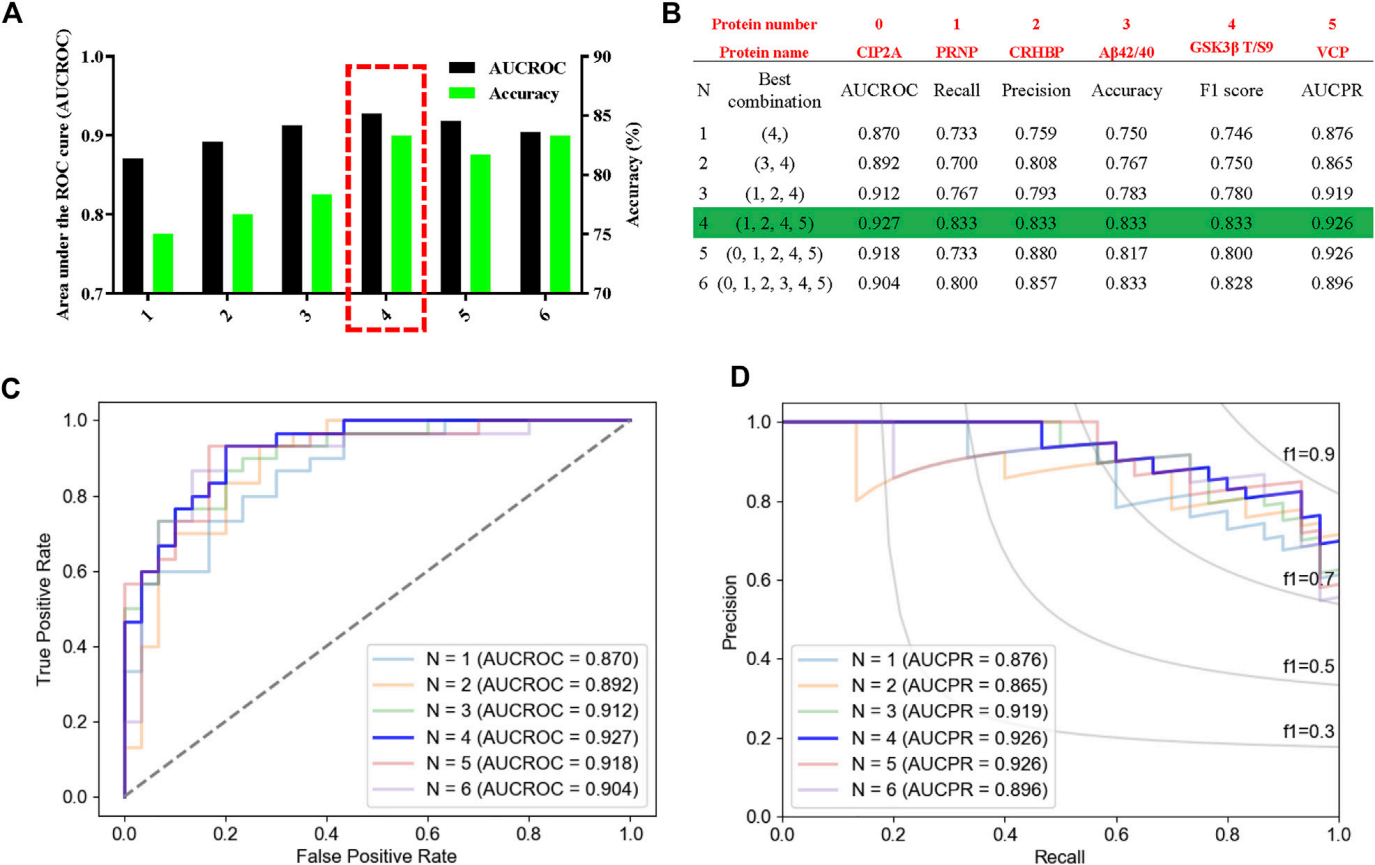
High performance discrimination of T2DM-MCI from T2DM-nMCI achieved by machine learning. **(A)** ROC area and corresponding accuracy based on the leave-one-out (LOO) algorithm in the validation set. The red box shows the selected protein with high area under the curve (AUC) and accuracy for the blinded test set. **(B)** Corresponding protein and various parameters for evaluating the efficiency of the biomarkers under each best combination. Red numbers represent the corresponding protein. **(C,D)** Based on the LOO algorithm, the area under the receiver operating characteristic curve (AUCROC) and precision–recall curve (AUCPR) for each best combination of biomarkers. AUCROC was based on the true-positive rate and false-positive rate: True-positive rate = [true-positive/(true-positive + false-negative)], False-positive rate = [false-positive/(true-negative + false-negative)]. PRAUC was obtained based on precision and recall: Precision = [true-positive/(true-positive + false-positive)]; Recall = [true-positive/(true-positive + false-negative)]. In addition, F1 score = 2 * (precision * recall)/(precision + recall).

Then, we used leave-one-out (LOO), a classic machine learning method, to build the most accurate and rigorous diagnostic model for cognitive decline in T2DM patients. By permutation and combination of each biochemical index, only one sample was retained as the verification set at each time, and the remaining samples were all used for the training set. Therefore, a total of 3,780 cycles of verification were carried out. After LOO full cross-validation, we found that the combination of PRNP, CRHBP, VCP, and rGSK-3β(T/S9) had the highest diagnostic efficiency with a maximum receiver operating characteristic (ROC) (AUCROC = 0.927), accuracy of 83.3%, recall of 0.833, precision of 0.833, F1 score of 0.833, and the precision recall curve (AUCPR) of 0.926 (Figures 7A–D; Supplementary Table S3). After this rigorous algorithm, rGSK-3β was identified as the most valuable single diagnostic biomarker, with an AUCROC of 0.870, accuracy of 75.0%, recall of 0.733, precision of 0.759, F1 score of 0.746, and AUCPR of 0.876 (Figures 7A–D), which was consistent with our previous large cohort studies (Xu et al., 2016).

## Discussion

The early diagnosis of AD has been an unsolved problem, which severely impedes new drug development. In addition to different types of neuropsychological scales that cannot avoid subjective bias from the operators, the current laboratory methods for AD diagnosis were mainly aimed at measuring Aβ deposition and tau hyperphosphorylation, such as measuring Aβ and tau in cerebrospinal fluid by ELISA, in the brains using MRI and PET (Frisoni et al., 2010; McKhann et al., 2011; Rice and Bisdas, 2017). However, due to technical and cost reasons, these methods are difficult for popularization. Additionally, it is always too late to cure when patients have already started to feel/show AD symptoms. Thus, finding fast, convenient, and cost-effective biomarkers in high-risk populations is important for a reliable early diagnosis of AD.

Patients with T2DM are at high risk to suffer from AD. Periphery plasma or platelet proteomics has unique advantages in biomarker screening (Bader et al., 2020; Wang et al., 2020). Here, we used TMT-LC-MS/MS techniques to analyze the plasma protein expression profiles in T2DM-MCI and T2DM-nMCI patients. Among 1,320 identified proteins, 752 showed good reproducibility and 53 DEPs were significantly different in two groups. Bioinformatics analysis showed that the DEPs were involved in multiple CNS-related diseases and amyloid neuropathies, and the changes in SERPINA1, PRNP, VCP, and APOC4 had a strong correlation to high-risk genes for AD. In addition, the deregulated glucose metabolism in diabetic patients was closely associated with FBP1 and ALDOB, the molecules involved in glycolysis/gluconeogenesis. By integrating proteomics and machine learning data, we identified that changes in PRNP, CRHBP, VCP, and rGSK-3β had the greatest power to identify cognitive decline in T2DM patients.

Amyloid neuropathies were remarkably enriched in the gene–disease network (DisGeNET). Our data showed that plasma Aβ42/40 was significantly increased in T2DM-MCI compared with T2DM-nMCI, which may be related to amyloid neuropathies protein CIP2A. As an endogenous PP2A inhibitor, CIP2A promotes tau and APP phosphorylation and Aβ production (Shentu et al., 2018). More generally, it is believed to promote the proliferation of cancer cells, the growth of non-adherent cells, and anti-apoptosis (Soofiyani et al., 2017). In addition, SERPINA1 that can bind with Aβ (Kouza et al., 2017) was significantly increased in T2DM-MCI compared with T2DM-nMCI. PRNP, also known as PrPc, mediates the neurotoxicity caused by Aβ, and conditional knockout of PRNP can reverse memory deficits and synaptic disorders in AD mice (Gimbel et al., 2010; Salazar et al., 2017). It should be noted that PRNP plays a role in reno-protective effects and kidney iron uptake and may also be a diagnostic marker for kidney-related diseases (Yoon et al., 2021). VCP is located in different organelles and plays important roles in autophagy, mitochondrial disorders, endoplasmic reticulum (ER)-stress, and DNA damage repair (Meyer et al., 2012). We found that the amyloid neuropathy-related proteins VCP, SERPINA1, and PRNP had strong connectivity with APP and other AD high-risk genes, such as MAPT, APOE, and PSEN1/2, in the protein interaction modules. VCP mutation decreases its enzyme activity, which retards the tau degradation pathway (Darwich et al., 2020).

Among the CNS disorder-associated proteins identified by proteomics, such as CRHBP, IGFBP3, PEPD, and GUSB, CRHBP was significantly increased in T2DM-MCI compared with T2DM-nMCI patients verified by ELISA. The level of CRHBP-mediated corticotropin-releasing factor (CRF) was significantly decreased in the cortex and cerebrospinal fluid of AD patients (Bissette et al., 1985; May et al., 1987); therefore, downregulating CRHBP may increase the level of free CRF, which could be an attractive direction for the treatment of AD (Vandael and Gounko, 2019). Indeed, a study on the AD-like animal model had shown that targeting CRHBP could improve brain functions with restoration of learning and memory (Behan et al., 1997). These data together suggest that the increase of CRHBP may contribute to cognitive decline in T2DM patients by inhibiting CRF.

Acidosis may increase the risk of dementia in T2DM patients (Chen et al., 2019). Our data showed that the glycolysis/gluconeogenesis-related proteins FBP1, ALDOB, and UQCRC2 were dysregulated and enriched in the metabolic acidosis process in T2DM-MCI patients. As a rate-limiting enzyme, FBP1 catalyzes the reaction of fructose 1,6-bisphosphate to fructose 6-phosphate, while the deficiency of FBP1 increases the levels of uric acid and hyperalaninemia with the consequence of metabolic acidosis or ketosis (Bijarnia-Mahay et al., 1993). ALDOB catalyzes the conversion of fructose 1, 6-diphosphate into glyceraldehyde-3P, and lack of aldehyde acetal leads to the accumulation of F1-P substrates (Bouteldja and Timson, 2010). UQCRC2 is a subunit of the mitochondrial electron transport chain complex III, which affects the electron transport process and ATP generation (Fernandez-Vizarra and Zeviani, 2018). Thus, the omics data reveal that the reduced FBP1 and ALDOB and increased UQCRC2 can contribute to cognitive decline in T2DM patients through the mechanisms involving deregulated glucose metabolism and acidosis.

Together, we revealed by proteomics and bioinformatics analyses that the peripheral plasma molecular alterations could reflect the CNS disorders, and four molecules (namely, CIP2A, PRNP, CRHBP, and VCP) were identified to be closely associated with AD pathologies. Further studies by ELISA and Peterson’s analyses validated that the upregulation of CIP2A, PRNP, and CRHBP and downregulation of VCP were indeed correlated to cognitive decline in T2DM patients. The final leave-one-out (LOO) cross-validation confirmed that PRNP, CRHBP, and VCP combined with elevated rGSK-3β could most effectively distinguish T2DM-MCI from T2DM-nMCI with an AUC of 0.927 and accuracy of 0.833.

## Data Availability

All data used to support the findings of this study are included within the article. Raw data used to generate the figures are available at the Proteome Xchange Consortium (http://www.proteomexchange.org) *via* the PRIDE partner repository with the dataset identifiers PXD027870.

## Supplementary material

Supplementary Table S1 Significantly increased and decreased proteins in T2DM-nMCI vs. Ctrl.

Supplementary Table S2 Significantly increased and decreased proteins in T2DM-MCI vs. T2DM-nMCI.

Supplementary Table S3 Under each combination, the corresponding protein and various parameters for evaluating the efficiency of the biomarkers.

## Abbreviations

T2DM, type 2 diabetes mellitus; MCI, mild cognitive impairment; T2DM-nMCI, T2DM without MCI group; T2DM-MCI, T2DM with MCI group; CHD, coronary heart disease; ApoE, apolipoprotein E; MMSE, Minimum Mental State Examination.
