# Supplementary material for: Discovering new peripheral plasma biomarkers to identify cognitive decline in type 2 diabetes
Source: Front Cell Dev Biol. 2022 Nov 24;10:818141. doi: 10.3389/fcell.2022.818141 (PMC9729784; doi:10.3389/fcell.2022.818141)
Supplement: Supplementary file 4 [file Table4.DOCX]

**Dear Prof. Andrei Surguchov:**

Thank you very much for giving us the opportunity to revise our manuscript entitled “**Discovering new peripheral plasma biomarkers to identify cognitive decline in type 2 diabetes**” (Manuscript ID: 818141). According to the very constructive suggestions, we have revised the paper. The point-by-point responses are listed as follow.

When we carefully checked the data again, we found that the candidate biomarkers included in the machine learning modeling should be CIP2A, PRNP, VCP, CRHBP, Aβ42/40, and rGSK-3β, rather than IGFBP3 and PEPD, which were not significantly different (please page 12, lines 26 to 27). We redrew Fig.7 and corrected the data in the whole paper. The final leave-one-out (LOO) cross-validation confirmed that PRNP, CRHBP, VCP combined with elevated rGSK-3β could most effectively distinguish T2DM-MCI from T2DM-nMCI with AUC of 0.927 and accuracy of 0.833 (Fig. 7A-D). After LOO-CV, rGSK-3β was still identified as the most valuable single diagnostic biomarker, with AUCROC of 0.870, accuracy of 75.0 %, recall of 0.733, precision of 0.759, F1 score of 0.746, and AUCPR of 0.876 (Fig. 7A-D).

Here we express our deep apology to the editors and corresponding reviewers, and carefully proofread the data in the full text.

**Independent Review Report, Reviewer 1**

The strength of the manuscript is that the authors identified several proteins involved in the cognitive changes in type two diabetic patients. These proteins which may be used as biomarkers include CIP2A, rAβ42/40, and rGSK-3β(T/S9). They have the greatest power to identify cognitive decline in type two diabetic patients. This is an important finding, because the early diagnostic of diabetes mellitus will help to establish correct and efficient treatment. The results seem to be valid, because the authors used a combination of several comprehensive techniques for first identifcation and then validation of biomarkers. The methods include high-throughput and highly sensitive TMT–LC–MS/MS proteomics, contemporary bioinformatics, machine learning and biochemical analyses.

The following limitations of the manuscripts should be corrected.

Introduction:

1 After the sentence “the number of T2DM patients is rapidly increasing and that can greatly contribute to the increased prevalence of AD” the authors should add the reference to a recent article:” Caveolin: A New Link Between Diabetes and AD. Cell Mol Neurobiol. 2020 Oct;40(7):1059-1066. doi: 10.1007/s10571-020-00796-4.

Respond: Thanks for your valuable suggestion, we have added the literature to the corresponding sentence (please page 4, lines 13 to 14).

2 “Therefore, non-hypothesis-driven approaches are needed for finding novel periphery biomarkers.” The sense of this sentence is unclear. The authors should either explain why they believe that non-hypothesis-driven approaches should be used or delete this sentence.

Respond: In fact, the pathologic mechanisms of AD and T2DM are extremely complex, and non-hypothesis-driven proteomics can be used to delineate the plasma protein expression profile of T2DM-MCI and identify the most valuable candidate biomarkers.

3 “inter-molecular correlations” the term meaning is unclear and should be explained in details.

Respond: “inter-molecular correlations” the term meaning is “correlation analysis between molecules”. In order to find the relationship between molecules effectively, correlation analysis between molecules is a common data analysis method in omics.

Methods

“All samples excluded traumatic brain injury, brain tumors, drug abuse, alcohol addiction, and psychiatric disorders.” This is a clumsy sentence. It should be written as follows: “Patients with traumatic brain injury, brain tumors, drug abuse, alcohol addiction, and psychiatric disorders were excluded from the study.”

Respond: Thanks for your valuable suggestion, we have modified the corresponding sentences in the newly revised manuscript (please page 6, lines 6 to 7).

“we use Agilent Human 14 / Mouse 3 Multiple Affinity Removal System Column to remove high-abundance proteins in plasma.” High-abundance proteins may absorb biomarker proteins so their elimination may remove important biomarkers.

Respond: Thank you for your reminder. One of the potential drawbacks of removal of abundant proteins from the plasma is the simultaneous removal of some less abundant proteins. In fact, plasma pre-fractionation has proved to be necessary since it reduces the sample complexity and helps enrich low abundance proteins by removing highly abundant proteins. However, it also creates a problem due to concomitant removal of non-targeted ones. Choosing a depletion system remains a trade-off between what we want to remove versus what gets lost. The effect of Multiple Affinity Removal System (MARS) on enrichment of sample is the next question to address and thus forms the immediate focus of our next work. Further study on this aspect would provide new insights after in-depth analysis of the depleted fractions (Yadav et al. 2011).

**References:** Yadav AK, Bhardwaj G, Basak T, Kumar D, Ahmad S, Priyadarshini R, Singh AK, Dash D, Sengupta S. 2011. A systematic analysis of eluted fraction of plasma post immunoaffinity depletion: implications in biomarker discovery. PLoS One.6:e24442. Epub 2011/09/21.

Discussion

It is unclear what the authors mean giving this long list of last names:

Bader, Geyer, Muller, Strauss, Koch, Leypoldt, Koertvelyessy, Bittner, Schipke, Incesoy, Peters, Deigendesch, Simons, Jensen, Zetterberg and Mann 2020, Wang, Dey, Chen, Li, Niu, Cho, Wang, Bai, Jiao, Chepyala, Haroutunian, Zhang, Beach and Peng 2020)

In the list of references they give a citation to Bader as follows: Bader JM, Geyer PE, Muller JB, Strauss MT, Koch M, Leypoldt F, Koertvelyessy P, Bittner D, Schipke CG, Incesoy EI, et al. 2020. Proteome profiling in cerebrospinal fluid reveals novel biomarkers of Alzheimer's disease. Mol Syst Biol. Jun;16:e9356. Epub 2020/06/03.

These references should be corrected as follows: Bader et al., 2020; Wang et al., 2020

Respond: Thank you for your reminder, we have corrected the references to standard format.

**Independent Review Report, Reviewer 2**

Authors presented the crucial 3 biomarkers for the differentiation of T2DM-MCI from T2DM-nMCI for the early diagnosis and prevention.

1st: the criteria for diagnosing patients with T2DM was weak. Additional data on non-invasive HBa1C should have been presented to support the definite T2DM patients.

Respond: Thanks for your positive comments. Unfortunately, not all the people enrolled in our center were tested for HbA1c, and we will focus on HbA1c in future studies. In addition, type 2 diabetes diagnosis standard adopts the World Health Organization (WHO) (1999) criteria for diagnosis and classification of diabetes (Olokoba et al. 2012) (please page 5, lines 26 to 27). Typical symptoms of diabetes (polydipsia, polyuria, polyphagia, and weight loss) plus random plasma glucose ≥11.1 mmol/L, or FPG (Fasting plasma glucose) ≥ 7.0 mmol/L, or OGTT 2hPG (Oral glucose tolerance test 2-hour plasma glucose) ≥ 11.1 mmol/L.

**References:** Olokoba AB, Obateru OA, Olokoba LB. 2012. Type 2 diabetes mellitus: a review of current trends. Oman Med J. Jul; 27: 269-273. Epub 2012/10/17.

2nd: many of the indicated ELISA required different anticoagulants. Authors did not mention on the differential blood drawing methods from patients.

Respond: We draw blood from the elbow vein to the clinically standard EDTA·K2 anticoagulant tube , which can meet different ELISA testing.

3rd: Authors need to present the p-values of all biomarkers and AUC values.

Respond: Thanks for your positive comments. To address the reviewer’s concern, we have presented each combination, the corresponding protein and various parameters for evaluating the efficiency of the biomarkers in Supplementary Excel. 3. But LOO-CV is that only one sample was retained as the verification set at each time, and the remaining samples were all used for the training set, so unlike five-fold cross-validation, the p-values can be obtained.

4th: Authors need to cross-correlate the results from each category of different biomarkers by combining two or more proteins.

Respond: Thank you for the positive comments. To address the reviewer’s concern, we have presented each combination, the corresponding protein and various parameters for evaluating the efficiency of the biomarkers in Supplementary Excel. 3.

5rd: each category of different biomarkers should be discussed in AD and other complications of T2DM, like elevation of prion were found with patients with kidney problems.

Respond: As suggested, we have extended the discussion as follows:

It should be noted that PRNP plays a role in reno-protective effects and kidney iron uptake, and may also be a diagnostic marker for kidney related diseases (Yoon et al., 2021). More generally, it is believed to promote the proliferation of cancer cells, the growth of non-adherent cells, and anti-apoptosis (Soofiyani et al., 2017). VCP is located in different organelles and plays important roles in autophagy, mitochondrial disorders, endoplasmic reticulum (ER)-stress, and DNA damage repair (Meyer et al., 2012). (please page 14, lines 17 to 19, lines 23 to 27).

**References:** Yoon, S., Go, G., Yoon, Y., Lim, J., Lee, G., and Lee, S. (2021). Harnessing the Physiological Functions of Cellular Prion Protein in the Kidneys: Applications for Treating Renal Diseases. Biomolecules 11.

Soofiyani, S.R., Hejazi, M.S., and Baradaran, B. (2017). The role of CIP2A in cancer: A review and update. Biomed Pharmacother 96, 626-633.

Meyer, H., Bug, M., and Bremer, S. (2012). Emerging functions of the VCP/p97 AAA-ATPase in the ubiquitin system. Nat Cell Biol 14, 117-123.
